# Objectivizing issues in the diagnosis of complex rare diseases: lessons learned from testing existing diagnosis support systems on ciliopathies

**DOI:** 10.1186/s12911-024-02538-8

**Published:** 2024-05-24

**Authors:** Carole Faviez, Xiaoyi Chen, Nicolas Garcelon, Mohamad Zaidan, Katy Billot, Friederike Petzold, Hassan Faour, Maxime Douillet, Jean-Michel Rozet, Valérie Cormier-Daire, Tania Attié-Bitach, Stanislas Lyonnet, Sophie Saunier, Anita Burgun

**Affiliations:** 1 Centre de Recherche des Cordeliers, Sorbonne Université, INSERM, Université Paris Cité, Paris, F-75006 France; 2 HeKA, Inria Paris, Paris, F-75012 France; 3 Data Science Platform, Université Paris Cité, Imagine Institute, INSERM UMR 1163, Paris, F-75015 France; 4 Service de Néphrologie, Dialyse et Transplantation, Hôpital Universitaire Bicêtre, Assistance Publique-Hôpitaux de Paris (AP-HP), Kremlin Bicêtre, F-94270 France; 5 Laboratory of Renal Hereditary Diseases, Imagine Institute, INSERM UMR 1163, Université Paris Cité, Paris, F-75015 France; 6 Division of Nephrology, University of Leipzig Medical Center, Leipzig, Germany; 7 Laboratory of Genetics in Ophthalmology, Imagine Institute, INSERM UMR 1163, Université Paris Cité, Paris, F-75015 France; 8 Reference Centre for Constitutional Bone Diseases, laboratory of Osteochondrodysplasia, Imagine Institute, INSERM UMR 1163, Université Paris Cité, Paris, F-75015 France; 9 Service de médecine génomique des maladies rares, Hôpital Necker-Enfants Malades, AP-HP, Paris, F-75015 France; 10 Service d’Histologie-Embryologie-Cytogénétique, Hôpital Necker-Enfants Malades, AP-HP, Paris, F-75015 France; 11 Laboratory of Embryology and Genetics of Congenital Malformations, INSERM UMR 1163, Imagine Institute, Paris Cité, Paris, F-75015 France; 12 Department of Medical Informatics, Hôpital Necker-Enfants Malades, AP-HP, Paris, F-75015 France; 13 Universite Paris Cite, Paris, France

**Keywords:** Ciliopathy, Clinical decision support, Rare diseases, Electronic health record, Artificial intelligence, External evaluation, Human phenotype ontology, Early diagnosis, Patient similarity

## Abstract

**Background:**

There are approximately 8,000 different rare diseases that affect roughly 400 million people worldwide. Many of them suffer from delayed diagnosis. Ciliopathies are rare monogenic disorders characterized by a significant phenotypic and genetic heterogeneity that raises an important challenge for clinical diagnosis. Diagnosis support systems (DSS) applied to electronic health record (EHR) data may help identify undiagnosed patients, which is of paramount importance to improve patients’ care. Our objective was to evaluate three online-accessible rare disease DSSs using phenotypes derived from EHRs for the diagnosis of ciliopathies.

**Methods:**

Two datasets of ciliopathy cases, either proven or suspected, and two datasets of controls were used to evaluate the DSSs. Patient phenotypes were automatically extracted from their EHRs and converted to Human Phenotype Ontology terms. We tested the ability of the DSSs to diagnose cases in contrast to controls based on Orphanet ontology.

**Results:**

A total of 79 cases and 38 controls were selected. Performances of the DSSs on ciliopathy real world data (best DSS with area under the ROC curve = 0.72) were not as good as published performances on the test set used in the DSS development phase. None of these systems obtained results which could be described as “expert-level”. Patients with multisystemic symptoms were generally easier to diagnose than patients with isolated symptoms. Diseases easily confused with ciliopathy generally affected multiple organs and had overlapping phenotypes. Four challenges need to be considered to improve the performances: to make the DSSs interoperable with EHR systems, to validate the performances in real-life settings, to deal with data quality, and to leverage methods and resources for rare and complex diseases.

**Conclusion:**

Our study provides insights into the complexities of diagnosing highly heterogenous rare diseases and offers lessons derived from evaluation existing DSSs in real-world settings. These insights are not only beneficial for ciliopathy diagnosis but also hold relevance for the enhancement of DSS for various complex rare disorders, by guiding the development of more clinically relevant rare disease DSSs, that could support early diagnosis and finally make more patients eligible for treatment.

**Supplementary Information:**

The online version contains supplementary material available at 10.1186/s12911-024-02538-8.

## Background

There are approximately 8,000 rare diseases that affect about 400 million people worldwide. Most clinicians have limited knowledge about these diseases [1]. Moreover, several of them are characterized by a very high clinical and genetic heterogeneity. All these factors lead to underdiagnosis, misdiagnosis or delayed diagnosis of rare diseases. In order to accelerate the diagnosis process, which is of major importance so that patients can have access to appropriate support, personalized care and can benefit from treatments, one solution consists in automatically extracting phenotypes from patients’ electronic health records (EHRs) [2] and developing algorithms to diagnose them based on their phenotypes. It has been shown that narrative documents are mainly used by clinicians to report symptoms and comorbidities [3, 4]. This is even more important for rare diseases where patients’ clinical histories are reported by clinicians in text, as illustrated recently for Myrhe [5] and Dravet [6, 7] syndromes. More generally, recent studies showed that text reports provide much more phenotypic information [8–10] than structured data for models predicting diagnosis. Considering this unstructured information within text reports for diagnosis purpose is of major importance for rare diseases as early diagnosis can improve the management and progression of the disease [8]. In a recent review [11], we showed that several efforts have been made to develop diagnosis support systems (DSSs) for rare diseases, which can be categorized into three groups based on the number of targeted diseases: one specific disease, a group of diseases and the whole spectrum of rare or genetic diseases. Until 2019, almost all systems relying on phenotypes were disease recommendation systems dedicated to all rare diseases that work as follows: (1) each rare disease is described by a set of phenotype concepts that correspond to the signs and symptoms of the disease, generally encoded with the Human Phenotype Ontology (HPO) [12]; (2) Possible diagnoses of a new patient are then scored by comparing the phenotypic description of the patient to such knowledge using similarity metrics; (3) The system then returns a list of diseases ranked by the similarity score for each patient. By consequence, the use of such a system is based on the assumption that the tested patient has a rare disease, and the objective is to identify the correct one. These systems can be used in clinical practice to provide diagnostic support to non-expert clinicians in simple cases or to help domain experts select patients of interest for further investigation in complex cases. However, such systems were not designed for automated large-scale detection, and their performances in the context of complex diseases with rapidly evolving and incomplete knowledge bases are unclear. Since 2020, systems using machine learning and targeting a single disease have started to be developed [13–20]. In contrast with the disease recommendation systems, these approaches consider large scale clinical databases containing both rare and common diseases and they rely on machine learning to derive models from the data and classify patients.

Among rare diseases, ciliopathies perfectly illustrate the potential value and issues raised by patient data availability to improve diagnosis accuracy. Ciliopathies, notably those due to defects in the primary cilium, are an expanding group of severe and rare monogenic disorders with an estimated prevalence of 1/2000. So far, more than 50 ciliary disorders linked to variants in about 180 established ciliopathy-associated genes implying both phenotypic and genetic overlaps have been reported [21]. As primary cilia are ubiquitous cellular organelles, their dysfunction can lead to a large spectrum of manifestations [22] affecting mainly the kidneys, eyes, brain, liver and bone [23], among which kidney dysfunction leading to end stage kidney disease is a major cause of morbidity and mortality. Moreover, recent studies have demonstrated that renal ciliopathies are largely underdiagnosed [24]. Indeed, the rarity of the disease combined with the important phenotypic and genetic heterogeneity [25] make ciliopathies easy to confuse with other rare and common diseases and very difficult to diagnose by non-specialized clinicians. Being able to diagnose ciliopathy patients as early as possible is of major importance, so that they can benefit from appropriate support and potential future treatments. For example, a potential treatment for renal ciliopathies has been recently investigated with promising results [26]. Considering DSSs to help clinicians for the diagnosis of ciliopathies by taking advantage of all the information buried in textual reports could be a way to find undiagnosed ciliopathy patients and alleviate diagnosis wandering.

In the present study, our objective is to test existing DSSs with patient phenotypic data derived from their EHRs from an academic children’s hospital and to assess their performances for the detection of ciliopathy patients. As no system has been developed yet for ciliopathies, we focus on the generic disease recommendation systems dedicated to all rare diseases. The challenges met through our analysis will be analyzed and discussed in the “Discussion” section, with the objective to derive lessons that could be of help for the design and development of future dedicated systems for rare diseases taking advantage of large-scale clinical databases. We rely on the framework provided by the American Medical Association in 2022 [27] to address issues like population representativeness through the inclusion of cases and controls, data quality, and explicitness. Such criteria are not only beneficial for ciliopathy diagnosis but also hold relevance for the wider biomedical informatics community, aiding in the enhancement of DSS for various complex rare disorders.

## Methods

### Databases and data encoding

The Necker Children’s Hospital is a French reference center for rare and undiagnosed diseases that hosts the Imagine Institute, a research center specializing in genetic diseases. The clinical data warehouse developed by Necker/Imagine, named Dr Warehouse [28], contains more than 9 million documents from more than 800,000 patients from Necker hospital. Within EHRs, unstructured clinical notes are used by clinicians to describe clinical signs and detailed histories of their rare disease patients and, therefore, they provide valuable resources for diagnosis purposes [29]. The high-throughput phenotyping module of Dr. Warehouse enables the automatic extraction of all types of clinical entities from EHRs, including phenotypes and diseases based on the Unified Medical Language System (UMLS) [30].

Additionally, a database dedicated to ciliopathies, named Cilio-base, aggregates structured curated information for ciliopathy patients from different clinical departments of Necker Hospital and/or genetic departments of Imagine research Institute, including diagnoses (coded by experts from Necker/Imagine using the Orphanet nomenclature) and causal genes. More than 1800 patients with proven or suspected ciliopathy disorders are included, and 1100 of them have bi-allelic variants in one causative gene identified. 215 patients from the Cilio-base were followed at Necker Children’s Hospital, i.e., had clinical records in Dr Warehouse. We focused on these 215 patients.

### Patient selection

Ciliopathies are pleiotropic diseases and causal genes remain unknown for a significant number of cases. Only half of the Cilio-base patients were completely characterized with an identified causative gene and a precise clinical diagnosis, and this proportion remained the same when considering only the 215 patients from Cilio-base with EHRs in Dr Warehouse. For this reason, two datasets of cases were considered: (1) *cilio_clear*, for patients with a proven ciliopathy (identified pathogenic variants and a precise diagnosis) and (2) *cilio_fuzzy*, for patients with a suspected ciliopathy, i.e., with clinical features compatible with a ciliopathy but without pathogenic variant identified. For both datasets, patients were randomly selected, with the additional constraint for *cilio_clear* to cover all ciliopathy diagnoses present in patients followed at Necker hospital.

To assess the ability of the DSSs to differentiate ciliopathy patients from other patients, we included control patients from Dr Warehouse. We did not simply take a random sample of non-ciliopathy patients from Dr Warehouse, because testing the capacity of the DSSs to differentiate ciliopathy patients from patients who are completely different from ciliopathy is pointless in real-world clinical settings. As a common morbidity across several ciliopathies is renal function deterioration, we defined control patients as patients who exhibited some overlapping phenotypes with ciliopathy patients, namely, kidney defects. We reused the set of 10,462 “other-nephropathy” patients in Dr Warehouse defined in our previous study [31], i.e., nonciliopathy patients having at least one automatically extracted UMLS phenotype concept subsumed by the term kidney diseases ([C0022658]) in their EHRs.

We selected from this collection of control patients with nephrology related signs:

- a first control dataset (named *control_random)* of randomly selected patients matched on age (at the date of the most recent EHR file) and number of HPO phenotypes with selected cases.
- a second set of patients found similar with ciliopathy patients based on the patient-patient similarity methods developed in previous studies [31, 32]: the top 30 patients from the “other-nephropathy” patients who were the most similar with ciliopathy patients were reviewed by experts, and among them a total of 11 patients who were confirmed as non-ciliopathy patients were integrated into this second dataset (named *control_similar*).

All patient profiles were reviewed by ciliopathy experts from Necker/Imagine (SS, MZ, KB, FP, LH, TA-B) to validate their respective categories.

For each patient, the UMLS phenotype concepts extracted from his/her EHR via Dr Warehouse were converted to HPO terms using the mapping provided by the HPO consortium (HPO format-version: 1.2; data-version: releases/2019-11-08; downloaded on 2020-02-11). Phenotypes that could not be automatically converted were discarded. Before conversion to HPO, concepts directly associated with ciliopathy diagnosis (e.g., nephronophthisis) or genes (e.g., *NPHP1*) were removed. To ensure that each tested patient was followed at Necker Hospital with sufficient information in his/her EHR to characterize his/her condition, we focused on patients with at least 4 HPO concepts.

### Characteristics of the DSSs

Most systems dedicated to rare diseases [11] use phenotype concepts encoded with HPO [12], a patient-disease similarity-based method and return a ranked list of possible diseases. With a restriction to online accessibility and functionalities allowing the easy export of the results, three systems were considered for testing: Phenomizer [33], Genetic Diseases Diagnosis based on Phenotypes [34] (GDDP) and PubCaseFinder [35].

For Phenomizer, the authors developed a statistical model assigning p values to the resulting similarity scores between a patient and a disease, which is then used to rank the candidate diseases. Phenomizer was evaluated on a simulated cohort considering different levels of noise. GDDP proposed new methods to prioritize diseases based on semantic similarities and ontological overlap. Performance was evaluated considering the correct diagnosis rate within the top k using different ranges of cut-off value k (e.g., top 10) on simulated patients and medical records. PubCaseFinder provides a disease ranking based on disease-phenotype associations extracted from both PubMed and Orphanet using a similarity measure based on Information Content. The system was evaluated on medical records by measuring the correct diagnosis rate within the top k.

The versions of the systems and websites used for this analysis are those publicly available in June 2020. For GDDP, for each patient, we entered the HPO terms manually as free text and they were translated into HPO codes by the system. For Phenomizer, for each patient, each HPO term was manually entered and validated by using the autocomplete algorithm provided by the system. For PubCaseFinder, for each patient, we imported a file containing the HPO codes. For output, all three tools allow the automatic export of ranking diagnoses encoded in Orphanet [36] (PubCaseFinder), OMIM [37] (PubCaseFinder, GDDP), or both (Phenomizer). As PubCaseFinder independently returns Orphanet- or OMIM-encoded lists of ranked diagnoses, we tested the two systems separately, referred to as PubCaseFinder_Orpha and PubCaseFinder_OMIM, respectively, in the following sections. Default parameters were used to assess the patients.

### Evaluation

We re-used patients’ EHR data to assess the ability of the disease recommendation systems to differentiate ciliopathy cases from controls. A list of ciliopathies based on Orphanet (version 2.9.1) was established with all diseases that are descendants of the following Orphanet nodes: Ciliopathy (ORPHA:363,250), Ciliopathies with major skeletal involvement (ORPHA:93,426), and Joubert syndrome and related disorders (ORPHA:140,874). This list contains 72 distinct Orphanet codes mapped to 373 distinct OMIM codes and will be referred to as CIL-ORPHA (Additional Table 1) in the following text.

The three DSSs were evaluated in previous studies considering only rare disease patients with the objective to have the correct diagnosis of each patient as highly ranked as possible (i.e., measure of the overall correct diagnosis rate within the top k). Here, the situation is different: we included some control patients, and we are only interested in the diagnosis of one rare disease, i.e., ciliopathies. Consequently, the good performance of our tested DSSs is measured by:

- the ability to rank CIL-ORPHA diagnoses as high as possible for ciliopathy patients AND.
- the ability to rank CIL-ORPHA diagnoses as low as possible for control patients.

We considered a classification with the ranked list provided by each DSS using the following decision criterion: given a cutoff value k, a patient was classified as *positive* if a CIL-ORPHA diagnosis was found within the top k and as *negative* otherwise.

Individual results at the patient level were then aggregated per group and DSS, and several synthetic scores were computed.

As our primary objective was to detect ciliopathy patients, we first computed the true positive rate at rank k (TPR@k), or sensitivity at rank k, defined as the proportion of ciliopathy cases classified as *positive*. We then assessed the specificity of the DSS by computing the false positive rate at rank k (FPR@k), defined as the proportion of controls classified as *positive*. We eventually plotted the receiver operating characteristic (ROC) curve, which synthesizes these two indicators.

### Statistics and implementation

Associations between age, the number of HPO phenotypes and the rank of the target diagnosis were assessed with the Spearman correlation coefficient. The distributions of the rank of the target diagnosis for men and women were compared by performing the Kolmogorov-Smirnov test. All analyses were implemented using the R platform [38] with the tidyverse and ROCR packages.

## Results

### Datasets

**Fig. 1:**
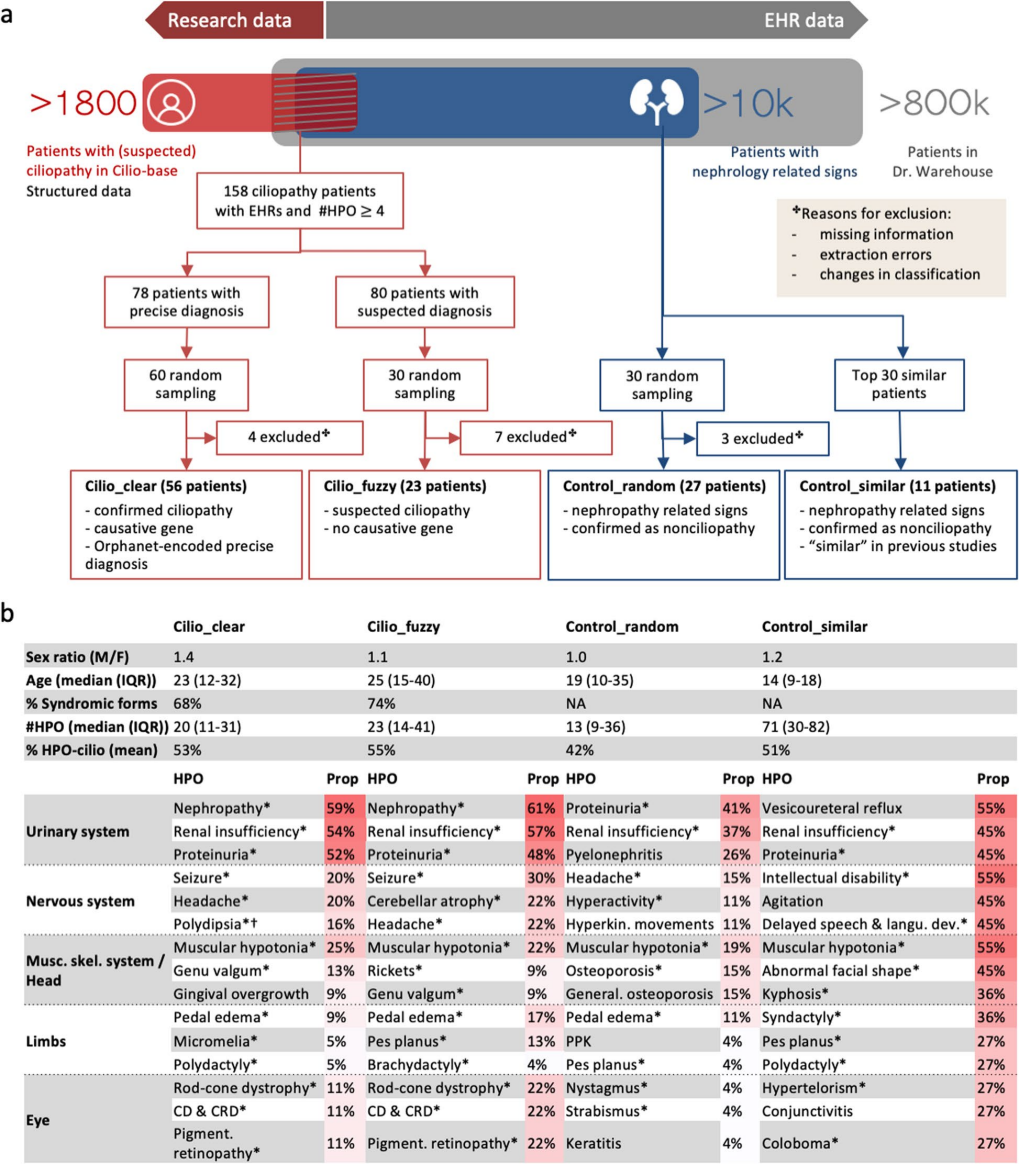
Case and control datasets: pipeline and description.** a** Schematic overview of the patient selection process. **b** Description of the datasets. For each patient, age corresponds to the age at the date of the most recent EHR file. For each dataset, the three most frequent HPO terms per class of disorders using the HPO hierarchy are presented. IQR, Interquartile range; CD & CRD, Cone/cone-rod dystrophy; PPK, Palmoplantar hyperkeratosis. *CIL-ORPHA-related HPO phenotypes. †Polydipsia is classified as a nervous system disorder in the HPO but is generally associated with urine concentration defect in the context of renal ciliopathies

The patient selection process and the corresponding numbers of patients are summarized in Fig. 1.

A total of 158 patients from Cilio-base had at least 4 HPO-encoded phenotypes in their EHRs, among whom 78 patients had a confirmed clinical and molecular diagnosis covering eleven distinct Orphanet codes of ciliopathy disorders. Among these 78 confirmed cases, we extracted a set of 60 patients covering all 11 ciliopathy Orphanet codes to build the *cilio_clear* data set. The distribution of diagnoses per database is provided in Additional Fig. 1. Regarding the *cilio_fuzzy* dataset, 30 patients were randomly selected from the 80/158 patients with suspected ciliopathies.

For the control groups, 30 patients were randomly selected as *control_random* matched for age and number of phenotypes with the cases. The eleven patients previously identified similar with ciliopathy patients were selected for the *control_similar* dataset.

An in-depth manual review by an expert was performed in order to keep only typical profiles in each class: four patients were excluded from *cilio_clear*, seven patients from *cilio_fuzzy*, and three patients from *control_random (*Fig. 1a), resulting in 79 ciliopathy patients and 38 controls.

The characteristics of the four datasets are shown in Fig. 1b. 86% of cases had renal impairment. 70% (55/79) of ciliopathy patients had multisystemic defects. For phenotype representation, patients in this study were associated with 1883 distinct UMLS phenotypes, and each patient was described with 64 terms on average. After conversion to the HPO, patients were associated with 792 distinct HPO terms, and each patient was described by 30 terms on average. UMLS terms that could not be mapped to HPO were either physiological characteristics that were not phenotypes in the HPO (e.g., systemic arterial pressure), or terms that were not sufficiently precise to be converted to HPO terms (e.g., cyst, fibrosis, hypertrophy). Ciliopathy patients and *control_similar* datasets had on average more than 50% of their HPO phenotypes associated with at least one CIL-ORPHA disease in the HPO, while patients in *control_random* had only 42% CIL-ORPHA-related HPO phenotypes. The most frequent HPO terms in all datasets were renal (e.g., renal insufficiency, proteinuria) and general (e.g., pain, fatigue) symptoms. Numerous neurological and skeletal disorders were found in *control_similar*. Disorders related to tubulointerstitial morphology were more frequent among cases than controls, and some features (e.g., polydipsia, cone-rod dystrophy) were specific to cases.

### Diagnosis performances

**Fig. 2:**
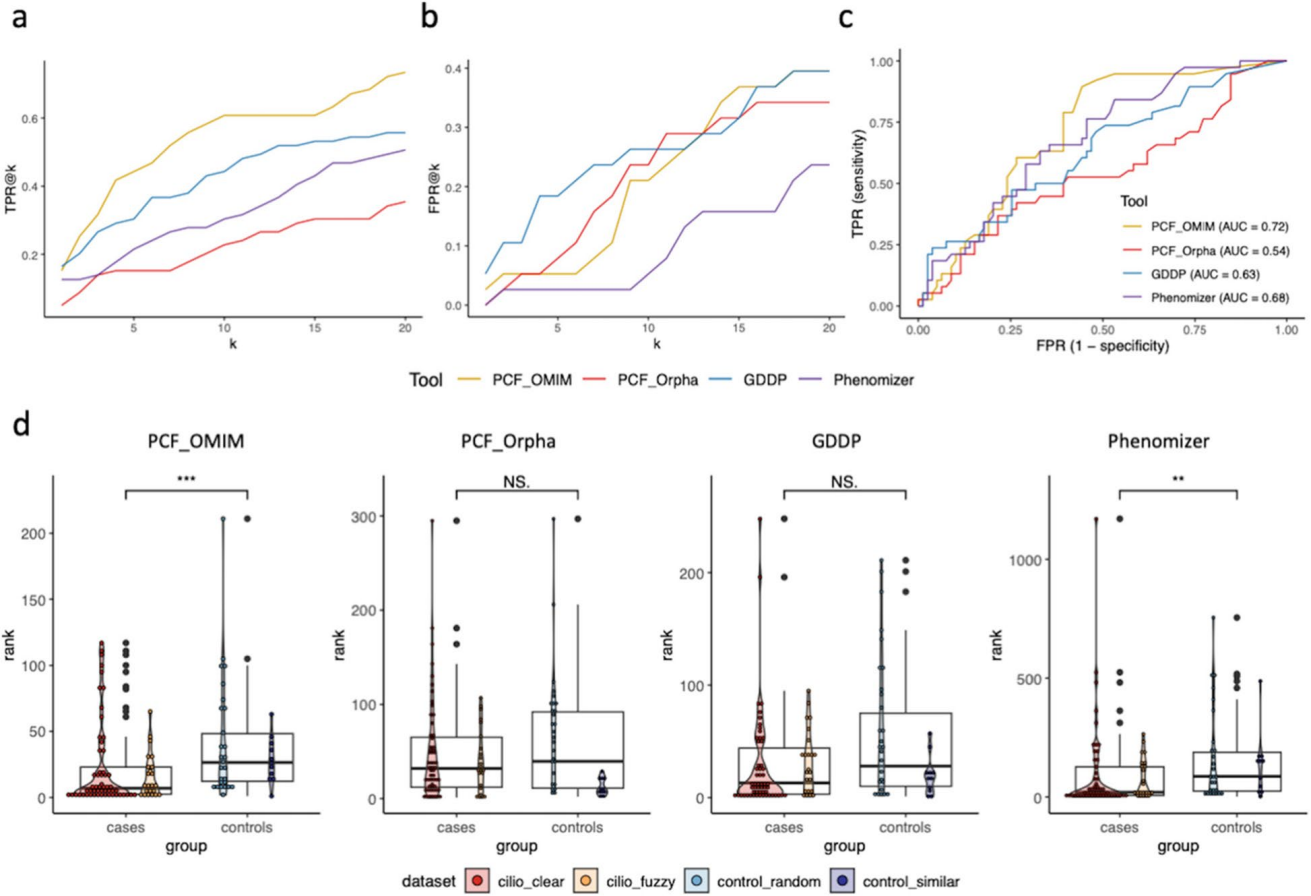
General performances of the DSSs.** a** and **b** represent the proportion of patients classified as having ciliopathy with different cutoff k values among the cases and controls, respectively. **c** ROC curves and AUCs for the four DSSs. **d** Distribution of ranks of the first CIL-ORPHA diagnosis for the four DSSs. The red dots (resp. blue) correspond to the ranks for the two datasets of cases (resp. controls). PCF, PubCaseFinder.

The general performances are summarized in Fig. 2. Regardless of the DSS, the rank of the first CIL-ORPHA diagnosis was influenced neither by age nor by the number of phenotypes. We first compared the TPRs and FPRs for values of k ranging from 1 to 20 (Fig. 2a., Fig. 2b.). PubCaseFinder_OMIM obtained the best TPRs but moderate specificity, while Phenomizer had the best specificity (lowest FPRs) but very low sensitivity. The synthesis of these two indicators, as obtained by the ROC curve (Fig. 2c.), showed that PubCaseFinder_OMIM exhibited the best area under the ROC curve (AUC) (0.72), followed by Phenomizer (0.68). We compared the distributions of the ranks between cases and controls for the four DSSs (Fig. 2d.) by applying the Kolmogorov-Smirnov test. The rank of the first CIL-ORPHA diagnosis was significantly lower for cases than for controls for PubCaseFinder_OMIM and Phenomizer but not for PubCaseFinder_Orpha and GDDP.

We identified five situations where all DSSs failed to select a CIL-ORPHA diagnosis:

1. Patients presenting with isolated symptoms were more difficult to diagnose than patients presenting with multisystemic symptoms (median rank = 12.5 vs. 5 for PubCaseFinder_OMIM).
2. Situations where some key phenotypes were missing or imprecise in the EHRs (e.g., “renal insufficiency” instead of “progressive renal insufficiency”).
3. Situations where some noisy phenotypes were present, and associated with incidental events (infection, diarrhea, fever, etc.).
4. Situations where the phenotyping algorithm only provided generic UMLS concepts without anatomical localization, such as “cyst” (C0010709) rather than the precise phenotypes, such as “renal cyst” (C3887499).
5. Some mappings between the UMLS and HPO were absent in the HPO source, e.g., for the UMLS term “Chronic kidney failure” (C0022661).

Table 1 provides a more detailed evaluation of the performances for the two DSSs with the best AUC, i.e., PubCaseFinder_OMIM and Phenomizer. Regarding cases, the TPRs were in general slightly better for *cilio_clear* than *cilio_fuzzy* with both the DSSs. PubCaseFinder_OMIM had the best TPR for k = 5, k = 10 and k = 20 for both datasets, and the performances for *cilio_fuzzy* were almost identical to those for *cilio_clear* (TPR@20 = 70% vs. 75%), showing that the system was able to detect ciliopathy even for patients with unknown pathogenic variants. In other terms, a TPR equal or higher than 70% could be achieved only with k = 20. Whatever the DSS, the TPR was always lower than 50% for k = 5 (ranging from 17 to 48%) for both *cilio_clear* and *cilio_fuzzy*. Regarding controls, the FPRs for low values of k were slightly higher for *control_similar* than for *control_random*. Phenomizer had lower FPRs for both datasets. As expected, the proportion of patients classified as *ciliopathy* was higher in the case datasets than in the control datasets for PubCaseFinder_OMIM and Phenomizer, which shows that, to some extent, these DSSs were able to distinguish between cases and controls, even controls exhibiting a high similarity score with cases. However, none of these systems obtained results good enough to be equivalent to “expert”.

**Table 1:** Performances of distribution of the ranks of the first CIL-ORPHA diagnosis

	# Patients	Performances			
		TPR@1	TPR@5	TPR@10	TPR@20
**Cilio_clear**					
PubCaseFinder_OMIM	56	**18%**	**48%**	**63%**	**75%**
Phenomizer	56	11%	23%	36%	52%
**Cilio_fuzzy**					
PubCaseFinder_OMIM	23	9%	**35%**	**57%**	**70%**
Phenomizer	23	**17%**	17%	17%	48%
		**FPR@1**	**FPR@5**	**FPR@10**	**FPR@20**
**Control_random**					
PubCaseFinder_OMIM	27	**0%**	4%	26%	41%
Phenomizer	27	**0%**	**0%**	**4%**	**26%**
**Control_similar**					
PubCaseFinder_OMIM	11	9%	**9%**	**9%**	36%
Phenomizer	11	**0%**	**9%**	**9%**	**18%**

TPR@k, true positive rate in the top k

### Differential diagnoses

As PubCaseFinder_OMIM obtained the best performances, especially for low values of k, we focused on this DSS to analyze the 10 diseases most frequently ranked within the top k (with k = 5) for cases and controls (Table 2). Among them, six were found for both cases and controls. Fabry disease shares a major feature, renal dysfunction, with ciliopathies and other nephropathies. The five other differential diagnoses in common were diseases affecting multiple organs and associated with a very important number of HPO terms: these diseases belonged to the top 1% of diseases in OMIM regarding the number of HPO phenotypes. Regarding cases, the only disease present in CIL-ORPHA was Alström syndrome, which was not represented among our study population but is also associated with a very high number of HPO terms. The other differential diagnoses for ciliopathy cases were all diseases with overlapping clinical features with ciliopathies, i.e., nephropathic cystinosis, congenital disorder of glycosylation, type 1a (CDG1A), and peroxisome biogenesis disorder 1 A (Zellweger).

**Table 2:** Ten most frequent diseases for cases and controls in the top 5 for PubCaseFinder_OMIM

Diseases in top 5 for cases	MIM id	# patients	Diseases in top 5 for controls	MIM id	# patients
Williams-Beuren syndrome^a^	194,050	40	Williams-Beuren syndrome^a^	194,050	27
Alstrom syndrome^b^	203,800	18	Rubinstein-Taybi syndrome 1^a^	180,849	11
Cystinosis, nephropathic	219,800	16	Smith-Lemli-Opitz syndrome	270,400	9
Fabry disease^a^	301,500	15	Fabry disease^a^	301,500	7
CDG1A	212,065	14	Cornelia de Lange syndrome 1^a^	122,470	6
Rubinstein-Taybi syndrome 1^a^	180,849	10	CHARGE syndrome^a^	214,800	5
Cornelia de Lange syndrome 1^a^	122,470	8	Bartter syndrome, type 2, antenatal	241,200	3
Costello syndrome^a^	218,040	8	Celiac disease, susceptibility to, 1	212,750	3
Zellweger syndrome	214,100	8	Coffin-Siris syndrome 1	135,900	3
CHARGE syndrome^a^	214,800	7	Costello syndrome^a^	218,040	3

^a^disease frequently suggested in the top 5 for both cases and controls

^b^disease belonging to the ciliopathy group

## Discussion

In this study, we evaluated the performance of current DSSs on complex genetic diseases, and used the example of ciliopathies, a group of complex pleiotropic disorders caused by cilia dysfunction. As no dedicated DSS has been developed yet, we evaluated generic rare disease DSSs for the diagnosis of ciliopathies using all phenotypes extracted from patient EHRs. The evaluation was performed in a children’s hospital specializing in genetic diseases but also serving as a general pediatric center for the local population.

In the original paper, Phenomizer [33] outperformed other scores on a cohort of simulated patients, ranking the correct diagnosis as the first proposal in more than 75% of the cases. GDDP [34] was compared with existing methods and outperformed them on medical records (top 10 diagnosis rate = ~ 32% for GDDP vs. ~ 4% for Phenomizer and ~ 20% for BOQA [39]). PubCaseFinder [35] was compared to other tools (Phenomizer and Orphamizer) on medical records and globally reached results comparable with Phenomizer. It obtained a top 10 diagnosis rate of 57% (Phenomizer = 47%, Orphamizer = 31%). These results highlight the variability of performance depending on the dataset under study. In the present study, PubCaseFinder_OMIM obtained the best rate of true positives for k = 20 (TPR > 70%) but the TPR scores dropped to 35–48% for k = 5. Moreover, it misclassified controls with an FPR@20 higher than 40%. Phenomizer obtained the lowest rate of false positives, but its sensitivity was not high enough to identify ciliopathy patients. Overall, PubCaseFinder_OMIM exhibited the best AUC (0.72). Not surprisingly, patients with multisystemic symptoms were generally easier to diagnose than patients with isolated symptoms. This may be partly because most patients with isolated symptoms had renal impairment, which generally presents with nonspecific features [40]. To summarize, none of these systems obtained results good enough to be equivalent to “expert”. Several interrelated lessons emerged from our evaluation, and we have attempted to encapsulate the following four key lessons for the future developers and users of rare disease DSSs.

The first lesson learnt was that these DSSs should ideally be integrated into the existing healthcare ecosystem, interoperable with the EHRs and capable of leveraging EHR data. This is of major importance because unstructured clinical notes in EHRs are unique sources of clinical information for diagnosis purposes, in particular for rare diseases [29]. Analysing the EHRs of two academic health institutions, Liu et al. [41] showed that the phenotypic coverage was much higher in clinical notes (about 36% of all phenotypic concepts in HPO) than in structured data (4%), for phenotypes found in at least 10 individuals. However, they stated that the EHR were rarely explored yet to generate rare disease-phenotype associations [41]. In this study, we benefited from access to patient EHRs as well as to an automated pipeline in order to generate HPO-based phenotypic descriptions of patients. However, whatever the DSS, the data had to be entered on a patient-by-patient basis into the DSS, and one of the evaluated tools required manual input and validation of each phenotype for each patient. We claim that such a time-consuming process is a major obstacle for large external evaluation of DSSs and hinders their large-scale use in clinical practice. Moreover, the disconnection from the EHR constraints on the use of data-driven approaches. The landscape of DSS has been transformed recently in many disciplines ranging from cancer [42, 43], to COVID [44] and sepsis [45], with machine learning models developed on multi-site EHR data. However, as stressed out by Schaaf et al. in their review of clinical DSSs for rare diseases [46], machine learning has been far less developed for rare diseases than in other medical fields, whereas the availability of EHR data provides an opportunity for developing algorithms that identify patients having a high probability to have a disease from large clinical data warehouses [13–20]. Ciliopathies perfectly illustrate the potential benefits of these new approaches, as it is of paramount importance to identify patients with suspected ciliopathies before the development of irreversible lesions as a potential treatment has been recently investigated with promising results. However, most rare disease research groups do not take advantage of EHR-based data driven approaches and stay focused on traditional disease recommendation systems.

The second lesson learnt was that it is important to validate the performances of DSSs in real-life settings involving clinicians and domain experts, which can benefit from the interoperability with the EHR system by providing cases and controls and enabling evaluators to test DSS in real life conditions. The American Medical Association recently highlighted that clinicians should bring critical insights on AI applications and should be involved in shaping AI’s role [27]. Similarly, Youssef et al. highlighted that real-world studies are a mandatory step to evaluate the deployed models’ usefulness [47]. We share this vision and claim that all DSSs should have external evaluation mimicking real-life, like in the studies from Weber et al. [44] or Adam et al. [45]. Indeed, the global performance of a system may be much lower in real-world settings than the scores achieved in the test set, as shown for example for common skin diseases [48, 49] and rare cardiomyopathy [14]. This is especially true when complex pipelines are needed to extract phenotypic information from EHRS. As described in our previous work, some tools are even only evaluated on simulated patients, whereas when tested on real patients some of them obtained performances that were much lower than on simulated ones [34, 50]. For example, as highlighted in this study, Phenomizer was tested for comparison with developed tools in numerous studies and obtained results that highly depended on the dataset under study [34, 35, 51–53]. This is particularly important for rare diseases where the question is: among the patients having renal symptoms, who is suspected to have ciliopathy and should have genetic testing, and could potentially benefit from treatment? In our study, cases were tested in contrast to carefully selected controls. In order to mimic real situations, the controls were patients having overlapping phenotypes with ciliopathy patients but not diagnosed with ciliopathy. We think that the inclusion of such kinds of controls is of major importance, as one key difficulty with rare diseases is to differentiate them from common diseases with overlapping profiles. However, until now, most of the generic DSSs have been evaluated only on rare disease patients.

The third lesson learnt was that high-quality data is crucial to make DSSs effective. Quality issues include both EHR data quality and NLP extraction quality. As key information may be present only as text in the EHR, Garcelon et al. [54] and Schaaf et al. [55] suggested developing adequate NLP methods to extract reliable and accurate information from unstructured text. Indeed, as pointed out by recent studies [56], EHR data may include incomplete records and inaccurate information. Missing or incomplete data is an obstacle to getting a comprehensive view of a patient’s medical history, while imprecise or noisy phenotype descriptions further complicate the precise capture and representation of a patient’s profile from clinical narratives using NLP techniques [57]. For example, in our study, the Named Entity Extraction pipeline sometimes failed at coordinating the location with the phenotype into a single term: it did not extract “renal cyst” from sentences like “the renal ultrasound confirmed the presence of cysts.‘’ “Renal” and “cyst” were extracted independently and the mapping to “renal cyst” (HP:0000107) was not completed. As for imprecise EHR phenotypes, such granularity issues may lead to inappropriate disease recommendations. In a recent work [58], we proposed a hybrid method combining a dictionary-based method with deep learning to enrich the set of UMLS terms. We trained and evaluated it on a ciliopathy characterized by skeletal abnormalities, Jeune syndrome and could strongly improve the detection of phenotypes from the EHRs. We plan to adapt such a method for other ciliopathies, starting with ciliopathies with renal impairment.

The fourth lesson is related to the relevance of the algorithm. All the tested DSSs have leveraged medical ontologies such as HPO to address granularity issues, and use the associations among phenotypes, genes and diseases provided by OMIM and Orphanet to deliver a list of possible diseases. All three are fine-grained terminologies designed for rare diseases [55]. However, such knowledge bases do not reflect the state-of-the-art knowledge in many rare-diseases domains, like e.g., ciliopathies, where research is still very active. We think that transparency regarding the knowledge incorporated in the algorithm, e.g., in our case, the class/set of ciliopathy disorders defined with the algorithm, is key for the correct interpretation of the algorithm results. Moreover, complex disease subtypes exhibit important phenotypic and genetic overlap. In our study, the analysis of the top-ranked differential diagnoses suggested by PubCaseFinder_OMIM showed that several diseases, e.g., Zellweiger syndrome, were easily confused with ciliopathy because they also affect multiple organs and have overlapping phenotypes with ciliopathies. The poor performance of the DSSs suggests that the complexity of ciliopathies requires integrating more expert knowledge specific to ciliopathies into the model. Otherwise, we also observed among the top-ranked differential diagnoses an overrepresentation of diseases associated with a large number of HPO phenotypes, e.g., Rubinstein-Taybi and Williams-Beuren syndrome. This reveals the need for fine-tuning the algorithms or adequate weighting/normalization in the computation of patient-disease similarity to better match the specific characteristics of the targeted patient population. A step further, we believe that methods based on patient-patient similarity [59] should be more interesting to support early diagnosis than those based on patient-disease similarity since the real-world patient data contain a wide range of complex information. Supervised machine learning methods are another way to leverage EHR data from diagnosed patients to detect undiagnosed patients, but they are usually limited to a single diagnosis, e.g., *NPHP1* pathogenic variants [60], and should be extended to broader disease coverage through multiclass models. To minimize bias that may be produced by individual variability, unsupervised methods that allow the integration of different types of information can be considered beforehand to identify clusters of ciliopathy patients [61]. A more sophisticated solution consists in using graphs to represent complex systems such as interactions between proteins, comorbidities between diseases, or healthcare knowledge [62]. In the rare disease field, they have been used to identify patients sharing similar phenotypes [63]. Adapted clustering methods have been proposed to study the graph structure and derive information from this representation [64, 65]. Such methods could help identify clusters of patients based on the graph representation. In order to make these change happen, the rare disease community will have to overcome the challenges related with the scarcity of the data [54, 66], utilize not only dedicated databases and registries but all data collected during routine healthcare processes – structured, narrative reports, genetic, etc. and favor new digital models able to combine expert knowledge and machine learning approaches.

## Conclusions

Clinicians need DSSs to support diagnosis of patients that have symptoms shared by both rare and common conditions. Existing disease recommendation systems do not consider common diseases, and, although they rely on state-of-the-art semantic methods and ontologies, their performance in diagnosing highly heterogeneous rare diseases does not reach expert levels. Challenges related to interoperability, algorithm transparency, clinical validation, data quality, ontology use, and context of application have been highlighted in this study. We conclude that the effectiveness of a DSS is influenced by the model as well as by how it is integrated within the EHR system. These lessons can guide the development of more effective and clinically relevant rare disease DSSs, that could support earlier diagnosis of rare disease patients and offer new perspectives in patient management.

### Supplementary Information

Additional file 1: CIL-ORPHA list. List of ciliopathy diseases used for the classification. The list is built based on the Orphanet hierarchy and disease names are encoded using the Orphanet nomenclature.

Additional file 2: Proportions of ciliopathy diagnoses per database. Proportions of diagnoses among patients with medical and genetic diagnosis for Cilio-base (in red), Cilio-base ∩ Dr. Warehouse (in purple) and *cilio_clear* patients (in yellow).

## Data Availability

The clinical datasets from this study are not publicly available, as institutional officials expressed concern about the inability to guarantee anonymity. Aggregate data and datasets containing coarse-grained phenotypes are available upon request to the corresponding author.
