# Supplementary material for: Objectivizing issues in the diagnosis of complex rare diseases: lessons learned from testing existing diagnosis support systems on ciliopathies
Source: BMC Med Inform Decis Mak. 2024 May 24;24:134. doi: 10.1186/s12911-024-02538-8 (PMC11127295; doi:10.1186/s12911-024-02538-8)
Supplement: Supplementary file 2 — Additional file 2: Proportions of ciliopathy diagnoses per database. Proportions of diagnoses among patients with medical and genetic diagnosis for Cilio-base (in red), Cilio-base ∩ Dr. Warehouse (in purple) and cilio_clear patients (in yellow). [file 12911_2024_2538_MOESM2_ESM.pdf]

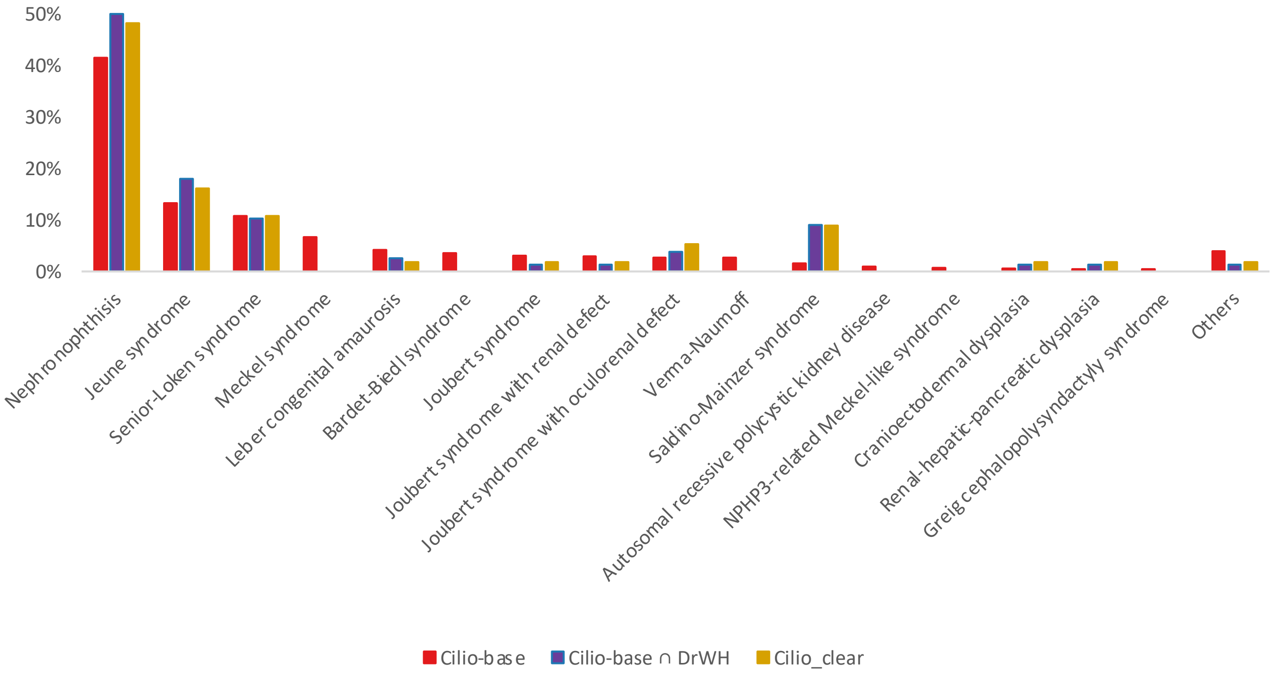

Additional Figure 1. Proportions of ciliopathy diagnoses per database. Proportions of diagnoses among patients with medical and genetic diagnosis for Cilio-base (in red), Cilio-base ∩ Dr. Warehouse (in purple) and *cilio_clear* patients (in yellow).
